# Exploring the novel SNPs in neuroticism and birth weight based on GWAS datasets

**DOI:** 10.1186/s12920-023-01591-y

**Published:** 2023-07-15

**Authors:** Xiao-Ying Zhou, Rui-Ke Liu, Chun-Ping Zeng

**Affiliations:** 1 Department of Endocrinology and Metabolism, The Fifth Affiliated Hospital of Guangzhou Medical University, Guangzhou, 510330 China; 2 Department of Endocrinology and Metabolism, SSL Central Hospital of Dongguan City, No.1, Xianglong Road, Dongguan, 523326 China

**Keywords:** Neuroticism, Birth weight, Conditional FDR, Genome-wide association study (GWAS)

## Abstract

**Objectives:**

Epidemiological studies have confirmed that low birth weight (BW) is related to neuroticism and they may have a common genetic mechanism based on phenotypic correlation research. We conducted our study on a European population with 159,208 neuroticism and 289,142 birth weight samples. In this study, we aimed to identify new neuroticism single nucleotide polymorphisms (SNPs) and pleiotropic SNPs associated with neuroticism and BW and to provide more theoretical basis for the pathogenesis of the disease.

**Methods:**

We estimated the pleiotropic enrichment between neuroticism and BW in two independent Genome-wide association studies (GWAS) when the statistical thresholds were Conditional False Discovery Rate (cFDR) < 0.01 and Conjunctional Conditional False Discovery Rate (ccFDR) < 0.05. We performed gene annotation and gene functional analysis on the selected significant SNPs to determine the biological role of gene function and pathogenesis. Two-sample Mendelian Randomization (TSMR) analysis was performed to explore the causal relationship between the neuroticism and BW.

**Results:**

The conditional quantile–quantile plots (Q-Q plot) indicated that neuroticism and BW have strong genetic pleiotropy enrichment trends. With the threshold of cFDR < 0.001, we identified 126 SNPs related to neuroticism and 172 SNPs related to BW. With the threshold of ccFDR < 0.05, we identified 62 SNPs related to both neuroticism and BW. Among these SNPs, rs8039305 and rs35755513 have eQTL (expressed quantitative trait loci) and meQTL (methylation quantitative trait loci) effects simultaneously. Through GO enrichment analysis we also found that the two pathways of positive regulation of “mesenchymal cell proliferation” and “DNA-binding transcription factor activity” were significantly enriched in neuroticism and BW. Mendelian randomization analysis results indicate that there is no obvious causal relationship between neuroticism and birth weight.

**Conclusion:**

We found 126 SNPs related to neuroticism, 172 SNPs related to BW and 62 SNPs associated with both neuroticism and BW, which provided a theoretical basis for their genetic mechanism and novel potential targets for treatment/intervention development.

**Supplementary Information:**

The online version contains supplementary material available at 10.1186/s12920-023-01591-y.

## Introduction

Neuroticism is defined as a personality trait linked to emotional instability, which is characterized by emotion dysregulation and negative affect [1]. Neuroticism is one of the major risk factors leading to psychological disorder such as major depressive disorder (MDD), and neuroticism is also related to some physical diseases, including type 1 diabetes and cardiovascular disease [2, 3]. In previous studies, neuroticism has been proven to be a heritable personality trait [4], and exploring the genetic factors of neuroticism may contribute to our understanding of genetic variation in psychiatric disorders.

Birth weight (BW) is a clinical indicator of predicted future growth and developmental problems [5, 6], and infants born with weight lower than 2500 g are considered low birth weight (LBW) infants [7]. According to World Health Organization (WHO) research data, the incidence of LBW is 17% worldwide, which is a vital public problem worthy of attention. Moreover, studies have shown that the occurrence of LBW increases the risk of neuroticism, and the risk of neuroticism increased with decreasing birth weight [7, 8]. Both neuroticism and BW are highly influenced by genetic factors, so we could explore whether there are common notable SNPs between neuroticism and BW with genome-wide association [9, 10].

Through GWAS, several single nucleotide polymorphisms (SNPs) of neuroticism or BW have been identified [10–12]. However only a small portion of genes and SNPs were found in neuroticism or BW due to insufficient statistical power or other reasons [13]. The conditional false discovery rate (cFDR) is an effective approach for identifying novel polygenic effects, and its strong statistical power can notably enhance the detection of shared SNPs in two independent complex phenotypes [14]. Furthermore, Mendelian randomization (MR) was used to determine whether exposure factors (such as gene expression) play a decisive role in outcome variables (such as complex traits or diseases) [15].

The aim of this study is to identify the potential functionally shared SNPs between neuroticism and BW by applying ccFDR analysis and new SNPs significantly associated with neuroticism and BW and to provide a theoretical basis for the genetic mechanism of the disease.

## Material and method

### Data sources

The GWAS summary data of neuroticism in this study were obtained from the CNCR/CTGlab (Center for Neurogenomics and Cognitive Research Complex Trait Genetics lab) released in 2017, including 14,978,477 SNPs from 449,484 European participants aged 39–73 years (https://ctg.cncr.nl/documents/p1651/sumstats_neuroticism_ctg_format.txt.gz) [16]. The GWAS summary data of birth weight were obtained using data from the Early Growth Genetics Consortium (EGG (Early Growth Genetics) Consortium (egg-consortium.org)) and UK Biobank released in 2017. The GWAS data include 13,891,969 SNPs from 298,142 European participants [12].

We conducted a validation study to increase the persuasiveness of this study. We selected data independent from the main study for validation analysis, which contains 143,699 European participants of birth weight [11] and 449,484 European participants of neuroticism [16].

We used data from the GWAS database to conduct MR analysis, in which the neuroticism dataset contains 374,323 samples (Trait: Neuroticism score—IEU OpenGWAS project (mrcieu.ac.uk)) and the birth weight dataset contains 261,932 samples (Trait: Birth weight—IEU OpenGWAS project (mrcieu.ac.uk).

### Data processing

GWAS data of neuroticism and BW were downloaded from the database, and we used the statistical software R (version 3.60) to cope with these data. We deleted the unnecessary variables and only retained the variables required for this study: SNP (RSID), Chr (chromosome), POS (NCBI build 37) and P (P value). First, we used the merge function to retain the common SNPs in both datasets and delete the SNPs only existing in a single trait data set. Second, given that there is an association between two alleles in the case of linkage disequilibrium, the frequency of simultaneous inheritance of the two genes is significantly higher than the original random frequency. Therefore, we used PLINK software to eliminate strongly associated SNPs with the HapMap 3 genotype as a reference. We performed LD-based pruning (r2 ≤ 0.2) and finally obtained 9,045,591 SNPs.

### Statistical analysis

We used conditional quantile–quantile plot (Q-Q plot) to visually verify whether two datasets come from the same distribution and to estimate whether the random variable obeys a known distribution. In the conditional Q-Q plot, the X-axis is the -log10[CDF(P)] value of the correlated SNPs of neuroticism on conditional phenotype BW, and the Y-axis is the -log10(P) value of the SNPs of the main function phenotype neuroticism. We stratified the P value from the function of significance associated with conditional traits p ≤ 1, p ≤ 0.1, p ≤ 0.01, p ≤ 0.001, p ≤ 0.0001. The greater the difference in the degree of deviation from different lines, the stronger the genetic pleiotropic enrichment between the two disease phenotypes.

Conditional false discovery rate (cFDR) has been widely used to identify novel genetic loci based on GWAS [17]. For the definition of false discovery rate (FDR), it can be understood as the probability that the SNP is not associated with the disease in the real situation when the P value of the hypothesis test of the association strength with disease is less than the pre-defined cut-off value. The purpose of FDR is to control the expected value of the proportion of "misidentified differential SNPs" to "identified differential SNPs" in multiple hypothesis testing, so as to identify as many true differential SNPs as possible. cFDR is to extend the FDR approach to be able to involve two phenotypes or diseases simultaneously. The specific formula for calculating the cFDR value of the association between the *i*th SNP and the main phenotype is:

$${\mathrm{cFDR}(a\mid b)}^{i}=\mathrm{P}\frac{i}{a} * \frac{\mathrm{Count}( {P}_{b}\le \mathrm{P}\frac{i}{ b}) }{\mathrm{Count}({P}_{a} \le \mathrm{P}\frac{i}{a} \& {P}_{b} \le \mathrm{p}\frac{i}{ b}i )}$$

If the P value of the hypothesis test was less than a pre-specified significance threshold, we considered this SNP is significantly associated with the main phenotype. In this study, when neuroticism is the principal phenotype and BW is the conditional phenotype, the cFDR is defined as cFDR (NE|BW), and vice versa cFDR (BW|NE).

We used the conjunctional conditional false discovery rate (ccFDR) to discover the genetic pleiotropic SNP both associated with neuroticism and BW. It is known that the ccFDR p-value is the larger cFDR p-value after the pleiotropy of the two traits is emerged. So if ccFDR is less than pre-specified significance threshold, it will be considered that this SNP is significantly correlated with neuroticism and BW, which is a genetic pleiotropic SNPs.

Conditional Manhattan plots usually used in GWAS to show the genetic SNPs which is notably associated the trait phenotype in the plot. In our study, Y axis was—log10 (P) of cFDR or ccFDR of SNPs, and a scatter represents a SNP site. So if the height of loci on Y axis is higher, the association with the trait phenotype is stronger.

Quantitative trait locus (QTL) refer to the position of SNPs controlling quantitative traits in the genome, and the DNA variation loci related to mRNA expression are called eQTL [18]. The meQTL may assist in identifying novel genes associated with disease and providing the connection between DNA sequence variation and phenotype [19]. We used BIOS QTL (https://www.genenetwork.nl/) [20, 21] to identify the expressed quantitative trait and eQTL analysis was performed to analyze the correlation between gene expression and genotype, in which genotype usually is transcribed RNA expression abundance.

### Pleiotropic SNPs function analysis

For gene annotation, we used functional analysis tools such as bioDBnet (bioDBnet—Biological Database Network (ncifcrf.gov) to annotate the significant functional SNPs [22]. Enrichment analysis was used to explore the function of the annotated SNPs based on the known gene database, including the specific pathway in which the SNPs were enriched in. Functional analysis plays a role in exploring the SNPs effects of on disease pathogenesis and we used GO (gene ontology terms database, KOBAS (bioinfo.org)) to conduct functional analysis [23]. To investigate the relationship between neuroticism and BW, we also conducted protein–protein interaction analysis using the STRING database (STRING: functional protein association networks (string-db.org)) [24]. We performed gene annotation of the SNPs associated with neuroticism and birth weight (cFDR < 0.001) and entered the annotated genes to get protein–protein interaction plots.

### Two-sample Mendelian Randomization (TSMR)

Mendelian randomization is used to assess the causal inference between modifiable exposure and clinically relevant outcome. There are three important prerequisites for MR analysis. First, the selected SNPs are highly associated with intermediate phenotypes or exposure factors. Second, the selected genes are not associated with confounders. Third, there is conditional independence between the selected genes and disease outcomes. When satisfying the above three conditions, we could explain that the gene is mediated by the intermediate phenotype and acts on the disease, and the intermediate phenotype or exposure can be inferred to be the cause.

In this study, we used SNPs as exposures to explore the causal relationship between neuroticism and BW. Five methods were used to evaluate the results. However, the data we used have removed the linkage disequilibrium and the heterogeneity and pleiotropy are negligible, so the Inverse Variance Weighted method is preferred to evaluate the result. Meanwhile we conducted forest plots and scatter plots to visualize the outcome. In the forest plot each horizontal black line reflects the results estimated by a single SNP using the Wald ratio method, so reasonable results can only be obtained by combining the results of all individual SNPs and the combined result is the bottom red line. In the scatter plot, the X axis is the SNPs on the exposure factor (BW), the Y axis is the SNPs on the outcome factor (neuroticism), and the color line shows the result of MR fitting.

## Result of cFDR and ccFDR analysis

### Assessment of pleiotropic enrichment

**Fig. 1:**
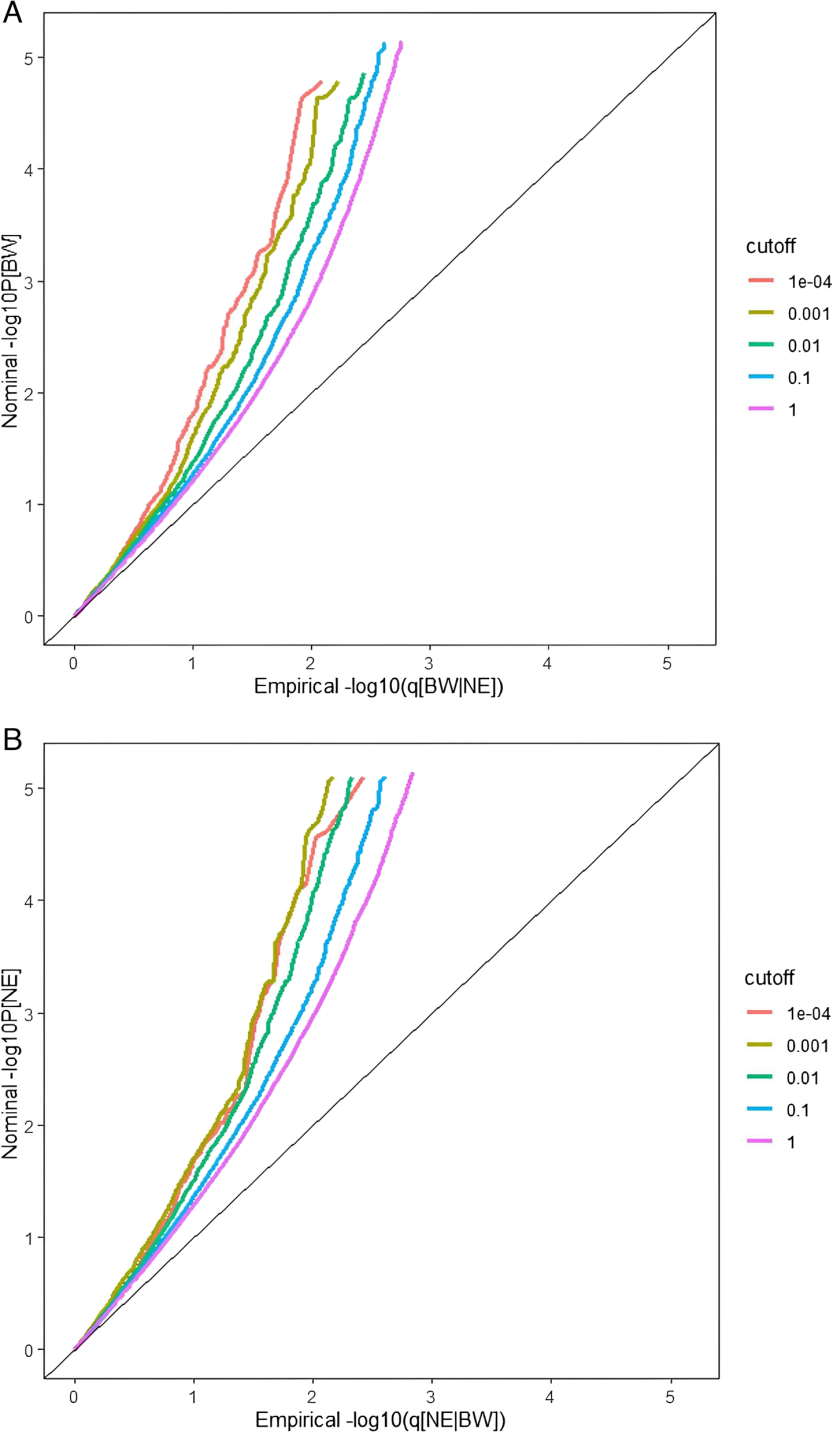
Stratified Q-Q plots. Neuroticism as function of the significance when BW as condition trait (**A**) and BW as function of the significance when neuroticism as condition trait (**B**)

We found that the curve deviates from the left as the corresponding *P*-value decreases in the Q-Q plot when neuroticism with BW as conditional phenotype (Fig. 1-A) and BW with neuroticism as conditional phenotype (Fig. 1-B). It indicates that a strong enrichment of genetic pleiotropy between the neuroticism and BW based on SNPs.

### Neuroticism or BW Loci Identified with cFDR

While the threshold of cFDR < 0.01, we identified 377 SNPs significantly related to neuroticism when BW as conditional trait. Even if used the more conservative threshold of cFDR < 0.001, we also identify 126 SNPs, which 2 SNPs among them have been identified in other studies [25, 26], and it means that most of the SNPs in neuroticism is first discovered in this study (ST1).

As for birth weight (BW), we identified 337 significantly related SNPs with the threshold of cFDR < 0.01. With the threshold of cFDR < 0.001, 172 SNPs of BW also have been identified, and 148 SNPs are novel loci firstly discovered in this study which others have been identified in previous GWAS study [27] (ST2).

**Fig. 2:**
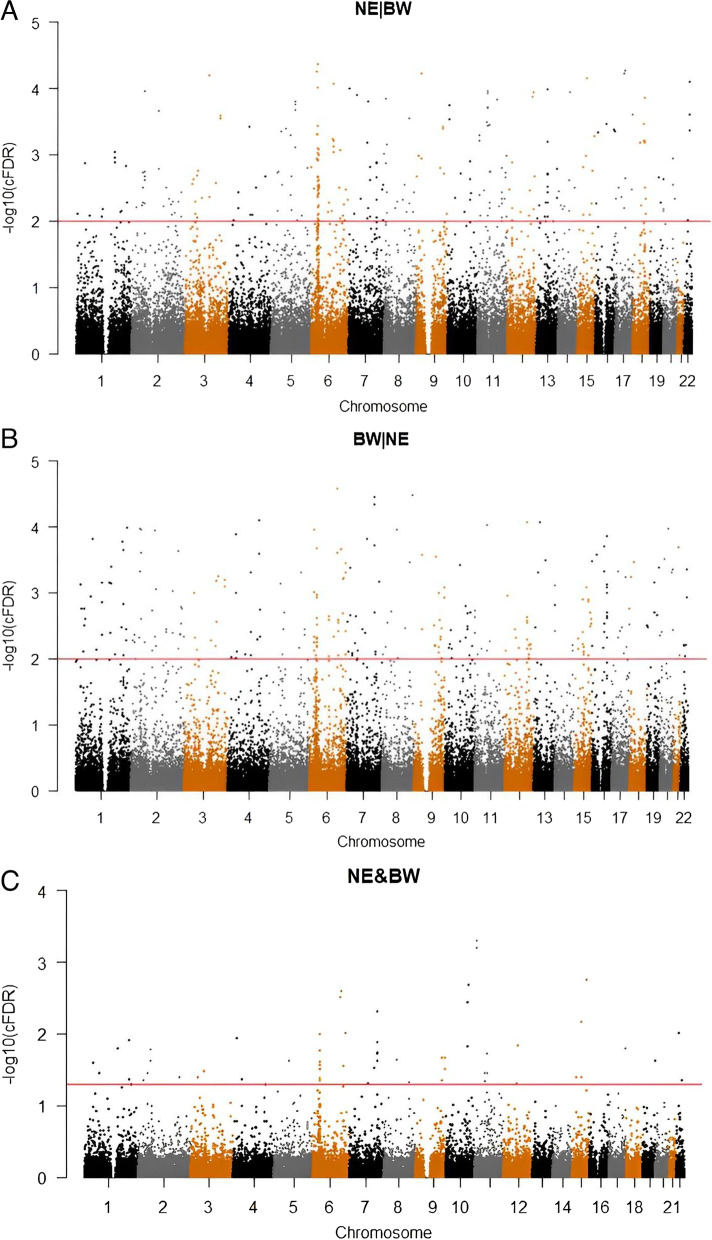
Manhattan plot. Conditional -log10(FDR) values for neuroticism given BW (NE|BW) (**A**), BW given neuroticism (BW|NE) (**B**), neuroticism and BW (NE&BW) (**C**)

We used manhattan plot to intuitively show the neuroticism related SNPs (Fig. 2) and BW related SNPs (Fig. 2). As shown in the figure, the y-axis represents the -log10 cFDR value of SNPs related to neuroticism or BW, and the x-axis represents the chromosome. The red horizontal line paralleled to the x-axis is the y-axis value when the cFDR value is 0.01 which the 377 significant SNPs of neuroticism and 337 significant SNPs of BW identified in this study are located above the red line, and the figure shows the chromosome which these SNPs mainly distributed in.

The identification of quantitative trait locus (QTL) plays an important role in discovering the regulatory functions of SNPs on phenotype. In these SNPs related to neuroticism we identify 99 SNPs have expression QTL (eQTL) effects, which two of them have 3’-UTR and splice acceptor function (ST1), and these SNPs may contribute to regulate gene expression. While in the SNPs related to BW, 152 SNPs have been identified as eQTL, and gene with 3'-UTR, 5'-UTR, synonymous and missense function may be relevant to regulate gene expression (ST2).

### Neuroticism and BW Common Loci Identified with ccFDR

We used the conjunction cFDR (ccFDR) method to identify pleiotropic genetic loci of neuroticism and BW. Finally, we identified 62 SNPs when ccFDR < 0.05, which 16 SNPs of them are relevant to neuroticism and 12 SNPs of them are relevant to BW (Table 1). The ccFDR manhattan plot was conducted to show SNPs distribution in chromosomes (Fig. 2). We identified these pleiotropic loci and found 51 SNPs have eQTL effects, and among these SNPs rs8039305 and rs35755513 had both eQTL and meQTL effects simultaneously, which rs5039305 have been identified associated with several mental illnesses including major depressive, bipolar disorder and schizophrenia [28].

**Table 1:** Conjunction cFDR value of 62 common SNPs in BW and neurotcism (ccFDR < 0.05)

SNP	CHR	POS	cFDR.BW	cFDR.NE	ccfdr	Gene Symbol	eQLT	meQTL	func annot
rs507288	1	37,203,702	0.001716	7.49E-09	0.001716	-	2	0	-
rs4660550	1	39,688,459	0.003108	0.025014	0.025014	MACF1	2	0	intronic
rs7534143	1	66,470,379	0.023308	0.034705	0.034705	PDE4B	3	0	intronic
rs6673081	1	1.55E + 08	4.87E-11	0.015693	0.015693	DCST2;ZBTB7B	34	0	3'-UTR
rs3737657	1	2.04E + 08	0.012323	0.006953	0.012323	ERLNC1;ETNK2	12	0	missense
rs7520276	1	2.06E + 08	0.04334	0.02542	0.04334	-	15	0	-
rs2339940	2	24,251,787	1.56E-07	0.044778	0.044778	WDCP	14	0	-
rs343969	2	44,956,905	0.034901	0.005843	0.034901	CAMKMT	2	0	intronic
rs848293	2	58,382,490	0.01665	0.000111	0.01665	VRK2	4	0	intronic
rs2863300	2	59,188,638	0.023732	0.002119	0.023732	LINC01122	0	0	intronic
rs4419186	2	1.49E + 08	0.012493	1.02E-06	0.012493	-	7	0	-
rs6430291	2	1.49E + 08	0.02889	4.89E-09	0.02889	MBD5	3	0	intronic
rs3771300	2	1.92E + 08	0.000926	0.040151	0.040151	STAT1	11	0	intronic
rs985463	3	34,110,517	0.040085	0.002747	0.040085	-	0	0	-
rs6795735	3	64,705,365	0.010544	0.032618	0.032618	ADAMTS9-AS2	2	0	intronic
rs1861044	4	15,539,498	0.009337	0.01138	0.01138	CC2D2A	3	0	intronic
rs4975032	4	39,687,955	0.042232	0.033192	0.042232	-	3	0	-
rs33419	5	71,846,721	0.017516	0.023526	0.023526	-	4	0	-
rs2523676	6	31,435,991	0.01705	0.00036	0.01705	DENND4B; MEN1	17	0	-
rs9267673	6	31,883,679	0.018701	0.024511	0.024511	C2	26	0	intronic
rs2734335	6	31,893,944	0.041245	9.59E-05	0.041245	C2	34	0	intronic
rs396090	6	32,977,535	0.043793	0.002408	0.043793	HLA-DOA	12	0	-
rs455567	6	33,252,115	0.030658	0.0029	0.030658	WDR46	26	0	intronic
rs6910233	6	33,534,726	0.026849	0.003178	0.026849	GGNBP1	7	0	-
rs13219530	6	33,657,493	0.00663	0.009883	0.009883	ITPR3	0	0	intronic
rs1262557	6	1.27E + 08	2.67E-05	0.003099	0.003099	-	0	0	-
rs9492432	6	1.3E + 08	0.002543	0.00084	0.002543	-	4	0	-
rs9376653	6	1.42E + 08	0.028039	0.010677	0.028039	-	0	0	-
rs1361024	6	1.52E + 08	0.002018	0.00958	0.00958	ESR1	2	0	intronic
rs13225695	7	86,702,298	0.049035	0.006419	0.049035	-	4	0	intronic
rs1868757	7	1.14E + 08	0.024787	0.029903	0.029903	FOXP2	0	0	intronic
rs10259338	7	1.27E + 08	0.023325	0.002149	0.023325	-	4	0	-
rs1057454	7	1.27E + 08	0.001957	0.004818	0.004818	ZNF800	4	0	5'-UTR
rs10258162	7	1.27E + 08	0.0189	0.001356	0.0189	LOC105375490	10	0	-
rs972088	7	1.27E + 08	3.57E-05	0.018052	0.018052	SND1	10	0	intronic
rs6969880	7	1.27E + 08	4.58E-05	0.013199	0.013199	SND1	2	0	intronic
rs10957133	8	61,300,510	0.018174	0.022692	0.022692	PDCL3P1;LINC01301	4	0	-
rs2737205	8	1.17E + 08	0.047282	0.000279	0.047282	TRPS1	2	0	intronic
rs1147322	9	1.26E + 08	5.52E-11	0.021735	0.021735	ZBTB6	12	0	5'-UTR
rs2540074	9	1.26E + 08	1.28E-10	0.044136	0.044136	STRBP	5	0	intronic
rs2078778	9	1.37E + 08	0.021297	0.00786	0.021297	-	7	0	-
rs12344192	9	1.37E + 08	0.019154	0.030832	0.030832	-	4	0	-
rs10786706	10	1.05E + 08	0.003589	0.002367	0.003589	WBP1L	22	0	-
rs4290163	10	1.05E + 08	0.015052	0.01028	0.015052	PCYT2; RPS8; ABCC8; TMEM53; SLC25A3	21	0	-
rs7092200	10	1.05E + 08	0.00207	0.001262	0.00207	CNNM2	7	0	-
rs10840346	11	10,062,999	2.44E-10	0.000621	0.000621	SBF2	3	0	intronic
rs4430500	11	10,254,371	3.92E-09	0.000508	0.000508	SBF2	5	0	intronic
rs3740699	11	47,504,075	0.046411	0.000109	0.046411	CELF1	0	0	intronic
rs7939420	11	47,723,938	0.035053	0.000197	0.035053	AGBL2	12	0	intronic
rs7117020	11	57,499,655	0.034854	0.006471	0.034854	TMX2-CTNND1; TMX2	4	0	intronic
rs4938893	11	58,095,004	9.41E-05	0.018753	0.018753	-	10	0	-
rs1988657	12	62,838,595	0.031134	0.049253	0.049253	-	2	0	3'-UTR
rs1042725	12	66,358,347	1.27E-54	0.014324	0.014324	HMGA2	2	0	3'-UTR
rs10161126	12	1.1E + 08	0.048314	1.13E-05	0.048314	-	13	0	-
rs11630273	15	40,878,978	0.039488	0.029126	0.039488	-	38	0	-
rs41434449	15	64,448,460	0.012405	0.039885	0.039885	PPIB; SNX22	0	0	3'-UTR
rs35755513	15	64,648,186	0.006669	7.05E-05	0.006669	CSNK1G1	2	Cis-meQTLs	splice acceptor
rs8039305	15	91,422,543	9.69E-10	0.001754	0.001754	FURIN	2	Cis-meQTLs	intronic
rs4075483	17	79,074,817	0.016128	0.000251	0.016128	BAIAP2	0	0	intronic
rs1017102	19	56,892,632	0.021051	0.023505	0.023505	ZNF582;ZNF542P	4	0	-
rs5753037	22	30,581,722	0.006136	0.009624	0.009624	HORMAD2; LOC105372988	9	0	-
rs4821981	22	41,415,644	0.044702	8.01E-05	0.044702	AC002378.1	18	0	-

### Pleiotropic SNPs functional analysis

To explore the biological function of the related annotation genes in the occurrence of the neuroticism, we conducted gene functional analysis (GO enrichment analysis) for the identified SNPs. For the SNPs related to neuroticism, the result showed that the SNPs mainly enrich in the pathways related to “glucose homeostasis” (*P* = 1.0*10–4), “type B pancreatic cell differentiation” (*P* = 1.1*10–4) and “neural crest cell differentiation” (*P* = 1.4*10- 4). As for the BW related SNPs, the GO result indicates that SNPs mainly enrich in “chromatin remodeling” (*P* = 1.0*10–4), “transcription factor binding” (*P* = 1.1*10–4) and “positive regulation of glucose import” (*P* = 1.1*10–4). Meanwhile these pathways are also related to RNA polymerase II such as negative regulation of transcription of RNA polymerase II and RNA polymerase II cis-regulatory region sequence-specific transcription, and hormone regulation such as insulin binding. The results showed that the two pathways of positive regulation of “mesenchymal cell proliferation” and “DNA-binding transcription factor activity” were significantly enriched in neuroticism and BW (Table 2).

**Table 2:** Gene ontology (GO) terms enriched for SNP-annotated genes with *P* ≤ 0.010 and common SNP gene with *P* < 0.016

Trait	#Term	Database	ID	Input number	Background number	Corrected *P*-Value	Input
nueroticism	glucose homeostasis	Gene Ontology	GO:0042593	4	115	0.00542848729402	MBD5|TCF4|PAX6|NCOA5
	type B pancreatic cell differentiation	Gene Ontology	GO:0003309	2	6	0.00564631175158	PAX6|MEN1
	neural crest cell differentiation	Gene Ontology	GO:0014033	2	7	0.00626592050451	MEF2C|FBXL17
	adenylate cyclase-inhibiting G protein-coupled glutamate receptor signaling pathway	Gene Ontology	GO:0007196	2	7	0.00626592050451	GRM8|GRM3
	actin cytoskeleton	Gene Ontology	GO:0015629	5	245	0.00626592050451	NCOA5|BAIAP2|SNCA|C2|MSRA
	regulation of synaptic plasticity	Gene Ontology	GO:0048167	3	50	0.00626592050451	MEF2C|BAIAP2|MAPT
	postsynaptic density	Gene Ontology	GO:0014069	5	251	0.00626592050451	DLGAP2|PCLO|DRD2|NSF|GRM3
	astrocyte differentiation	Gene Ontology	GO:0048708	2	8	0.00626592050451	PAX6|SOX6
	minor groove of adenine–thymine-rich DNA binding	Gene Ontology	GO:0003680	2	8	0.00626592050451	MEF2C|MAPT
	neuron-neuron synaptic transmission	Gene Ontology	GO:0007270	2	8	0.00626592050451	DLGAP2|DRD2
	postsynaptic modulation of chemical synaptic transmission	Gene Ontology	GO:0099170	2	9	0.00736508829015	DRD2|DCC
	cytoplasm	Gene Ontology	GO:0005737	21	4624	0.00794392457863	SBF2|BAG6|MAPT|RBFOX1|VRK2|RSRC1|PLCL2|CELF2|CELF1|MSRA|MEF2C|CSNK1G1|PAX6|ANKK1|SGCZ|FBXL17|SNCA|BAIAP2|MEN1|CKAP5|TAOK3
	RNA transport	Gene Ontology	GO:0050658	2	10	0.00794392457863	RBFOX1|CKAP5
	negative regulation of voltage-gated calcium channel activity	Gene Ontology	GO:1,901,386	2	10	0.00794392457863	DRD2|CRHR1
	negative regulation of transcription by RNA polymerase II	Gene Ontology	GO:0000122	8	832	0.00851319301933	TCF4|TRPS1|BBX|MEN1|MEF2C|PAX6|SNCA|SOX6
	SNARE binding	Gene Ontology	GO:0000149	3	64	0.00963922132356	SYT13|NSF|SNCA
	dynactin binding	Gene Ontology	GO:0034452	2	12	0.00963922132356	PAFAH1B1|MAPT
	protein localization to synapse	Gene Ontology	GO:0035418	2	12	0.00963922132356	BAIAP2|PCLO
BW	chromatin remodeling	Gene Ontology	GO:0006338	4	102	0.00521082186848	SMARCA4|RB1|TOP1|ESR1
	transcription factor binding	Gene Ontology	GO:0008134	6	325	0.00528915446255	ESR1|ENPP2|HMGA2|RB1|PIK3R1|SMARCA4
	positive regulation of glucose import	Gene Ontology	GO:0046326	3	38	0.00528915446255	MAPK14|PTPN11|PIK3R1
	negative regulation of transcription by RNA polymerase II	Gene Ontology	GO:0000122	9	832	0.00561702384388	STAT1|ESR1|CHD8|HMGA2|NOTCH1|RB1|NRIP1|RIPPLY3|SMARCA4
	RNA polymerase II cis-regulatory region sequence-specific DNA binding	Gene Ontology	GO:0000978	8	656	0.00588521152667	ZBTB6|STAT1|ESR1|HMGA2|NOTCH1|TOP1|NRIP1|SMARCA4
	insulin binding	Gene Ontology	GO:0043559	2	6	0.00593985997835	IGF1R|PIK3R1
	epithelial to mesenchymal transition	Gene Ontology	GO:0001837	3	43	0.00647443243419	LIMS1|HMGA2|NOTCH1
	positive regulation of metallopeptidase activity	Gene Ontology	GO:1,905,050	2	7	0.00713373065123	MAPK14|ANTXR1
	negative regulation of cold-induced thermogenesis	Gene Ontology	GO:0120163	3	47	0.00778605532871	NOTCH1|RB1|ADAM17
neurotcism&BW	positive regulation of mesenchymal cell proliferation	Gene Ontology	GO:0002053	2	23	0.0152210880734	FOXP2|STAT1
	DNA-binding transcription factor activity	Gene Ontology	GO:0003700	5	607	0.0152210880734	FOXP2|ZNF582|TRPS1|STAT1|ESR1

**Fig. 3:**
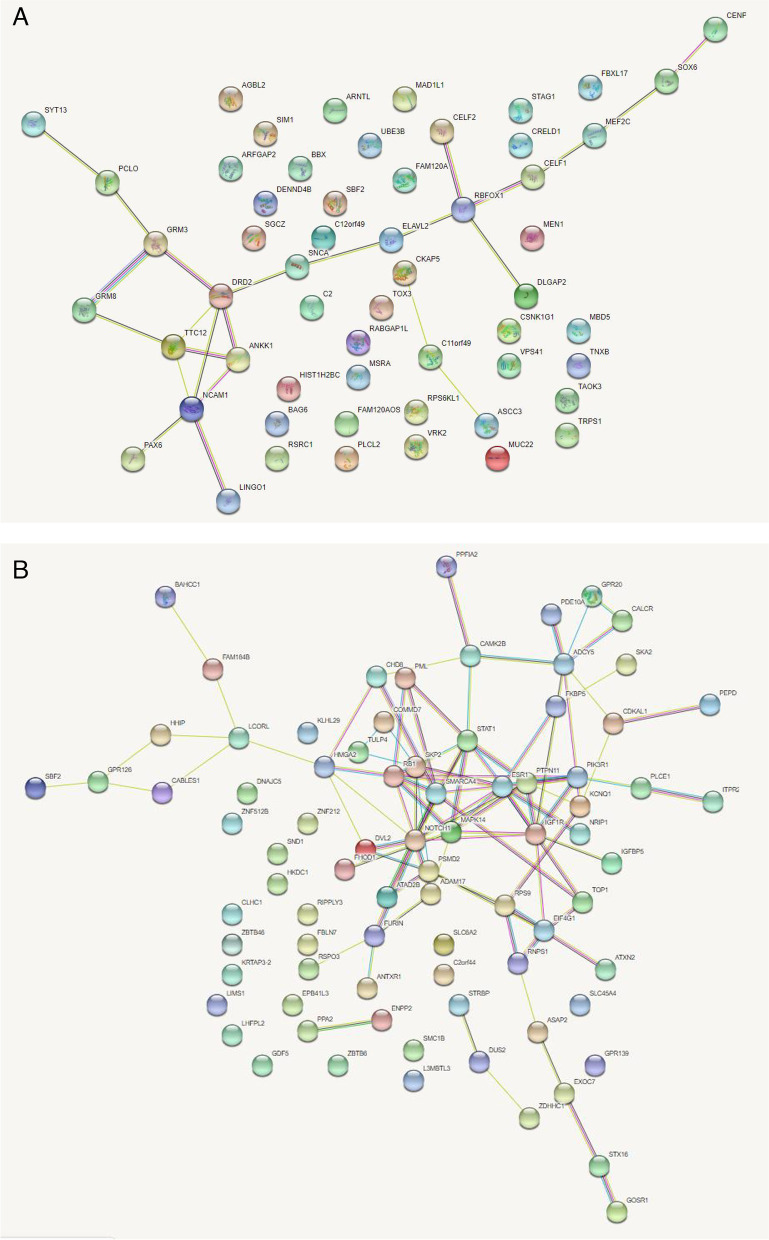
PPI plot. Protein-protein interactions between protein products of the corresponding gene in neuroticism (**A**) and BW (**B**)

We conducted protein–protein interaction (PPI) analysis by STRING 11.0. We entered the SNPs related to neuroticism and BW into the online STRING database and then come to a visual network plot, which may predict associations for a particular group of proteins (Fig. 3). Each solid circle in the PPI plot represents a kind of protein and the connection between them indicates that there is a certain connection or interaction, which different colors of the connection means different types of interaction. The association of neuroticism genetic expressed protein is weak, but DRD2, TTC12, ANKK1, NCAM1 and RBFOX1 play a key role in it. There is a strong connection between the annotation SNPs of BW in their biological function, especially SMARCA4, SKP2, RB1, MAPK14 and NOTCH1.

## Results of the validation study

**Fig. 4:**
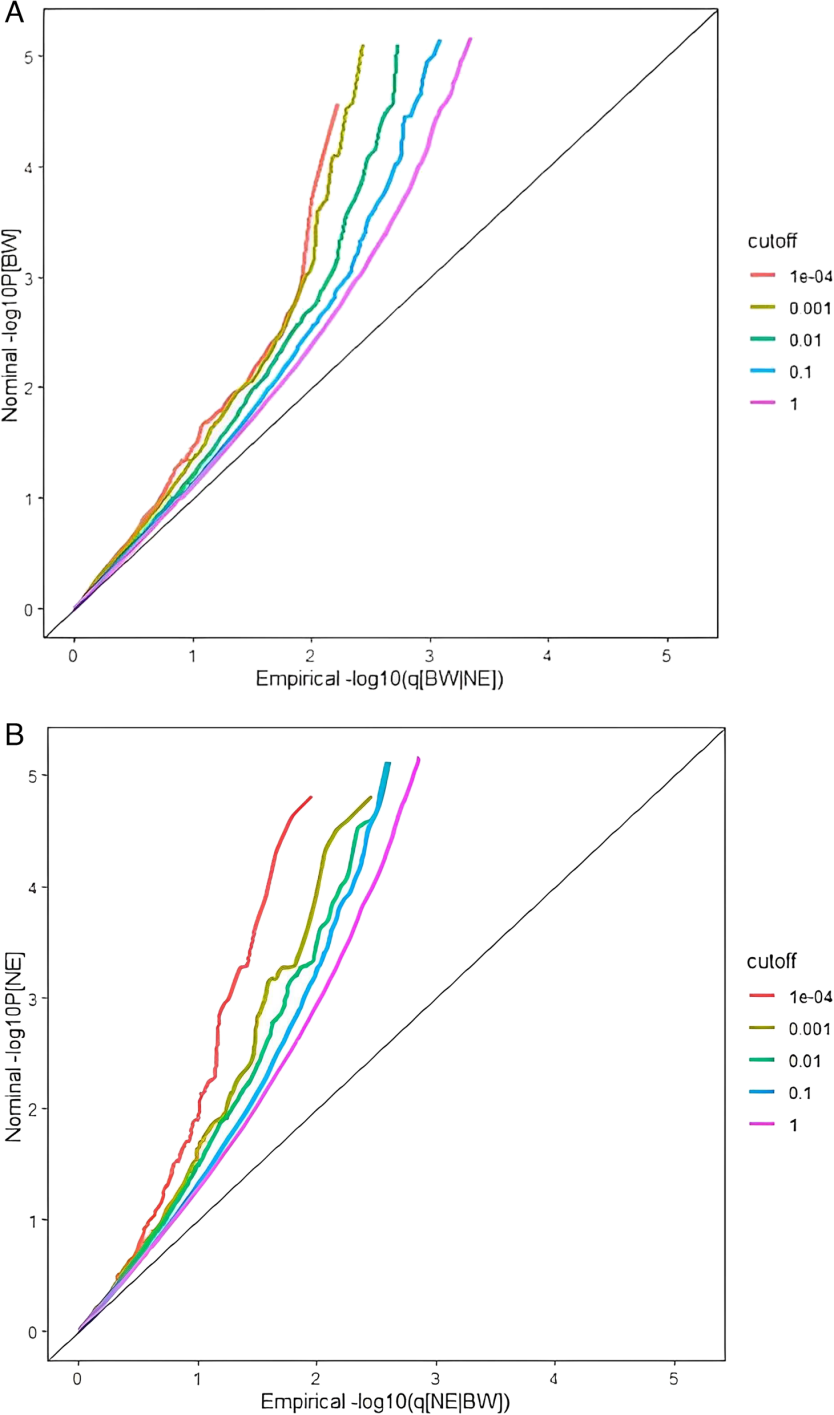
Stratified Q-Q plots. Result of the validation study that neuroticism as function of the significance when BW as condition trait (**A**) and BW as function of the significance when neuroticism as condition trait (**B**)

The conditional Q-Q plot (Fig. 4A/B) showed a significant deviation from the curves and it indicated that the two phenotypes has strong genetic pleiotropy. For neuroticism in condition of BW, we identified 317 SNPs, of which 117 SNPs are consistent with the main study analysis. Among the 70 SNPs significantly related to BW on condition of neuroticism, of which 52 SNPs are consistent with the result of the main study. As for the common SNPs of neuroticism and BW, we obtained 21 pleiotropic SNPs and 15 SNPs are consistent with the analysis results of the main study, so the association between neuroticism and BW is further confirmed in the validation analysis.

## Result of MR analysis

In the above study showed that there is genetic pleiotropy between neuroticism and BW, so we conducted MR analysis to explore the causal relationship between them. While BW as exposure factor and neuroticism as outcome factor, among 5 calculation methods including Mg Egger, Weighted median, Inverse variance weighted, Simple mode and weighted mode, all of their P-value are larger than 0.05 (Table 3).

**Table 3:** The result of neuroticism as exposure factor and bieth weight as outcome factor based on 5 MR methods

Exposure	Outcome	Method	b	se	*p*-value
birth weight|| id:ukb-b-13378	neuroticism|| id:ukb-b-4630	Mg Egger	0.0544336	0.14054115	0.6992039
birth weight|| id:ukb-b-13378	neuroticism|| id:ukb-b-4630	Weighted median	0.05579724	0.0576245	0.3328995
birth weight|| id:ukb-b-13378	neuroticism|| id:ukb-b-4630	Inverse variance weighted	-0.01830785	0.05320783	0.7307848
birth weight|| id:ukb-b-13378	neuroticism|| id:ukb-b-4630	Simple mode	0.06046687	0.14964102	0.6868617
birth weight|| id:ukb-b-13378	neuroticism|| id:ukb-b-4630	Weighted mode	0.10131709	0.10546998	0.3386408

**Fig. 5:**
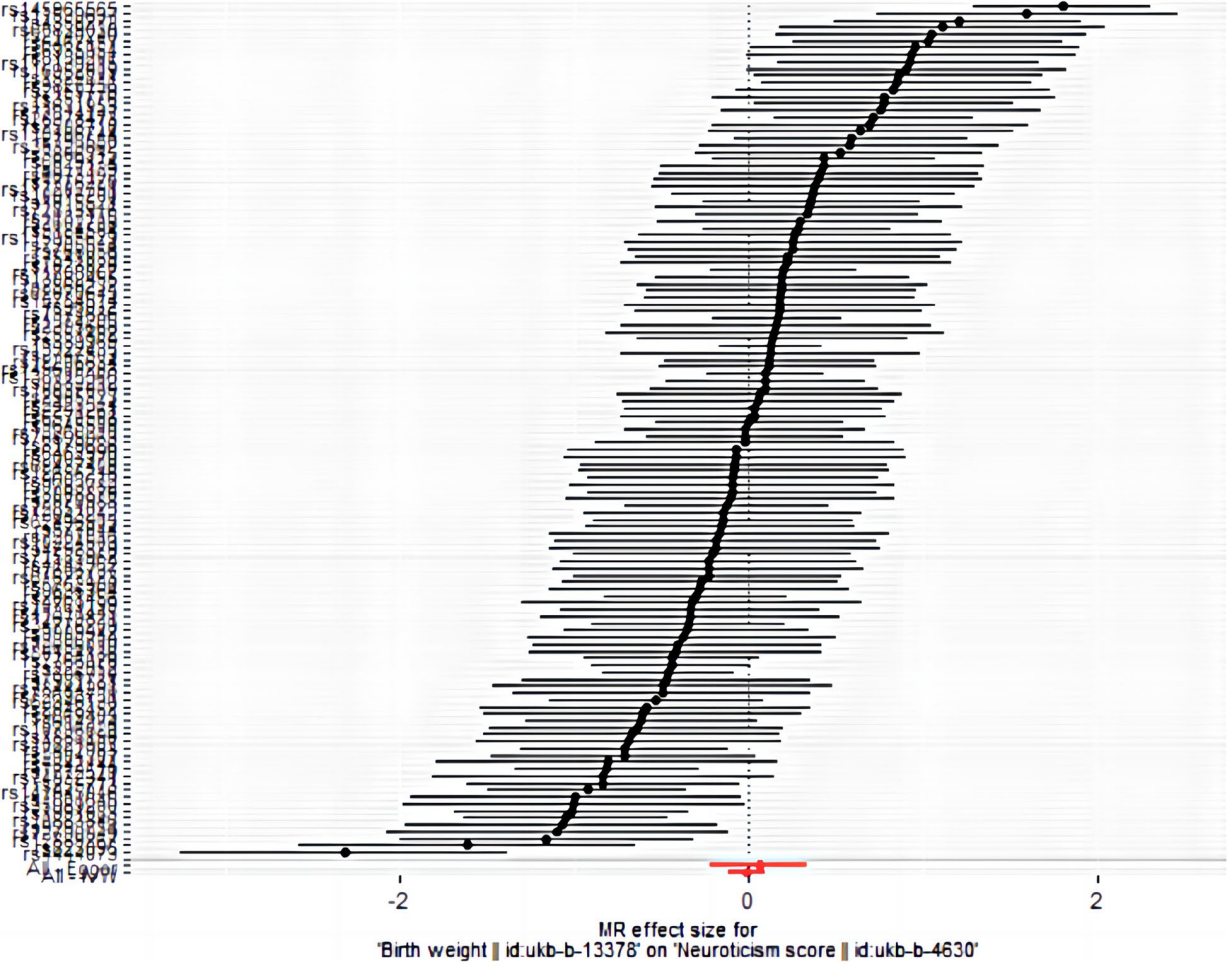
Forest plot. MR effect size for BW on neuroticism

**Fig. 6:**
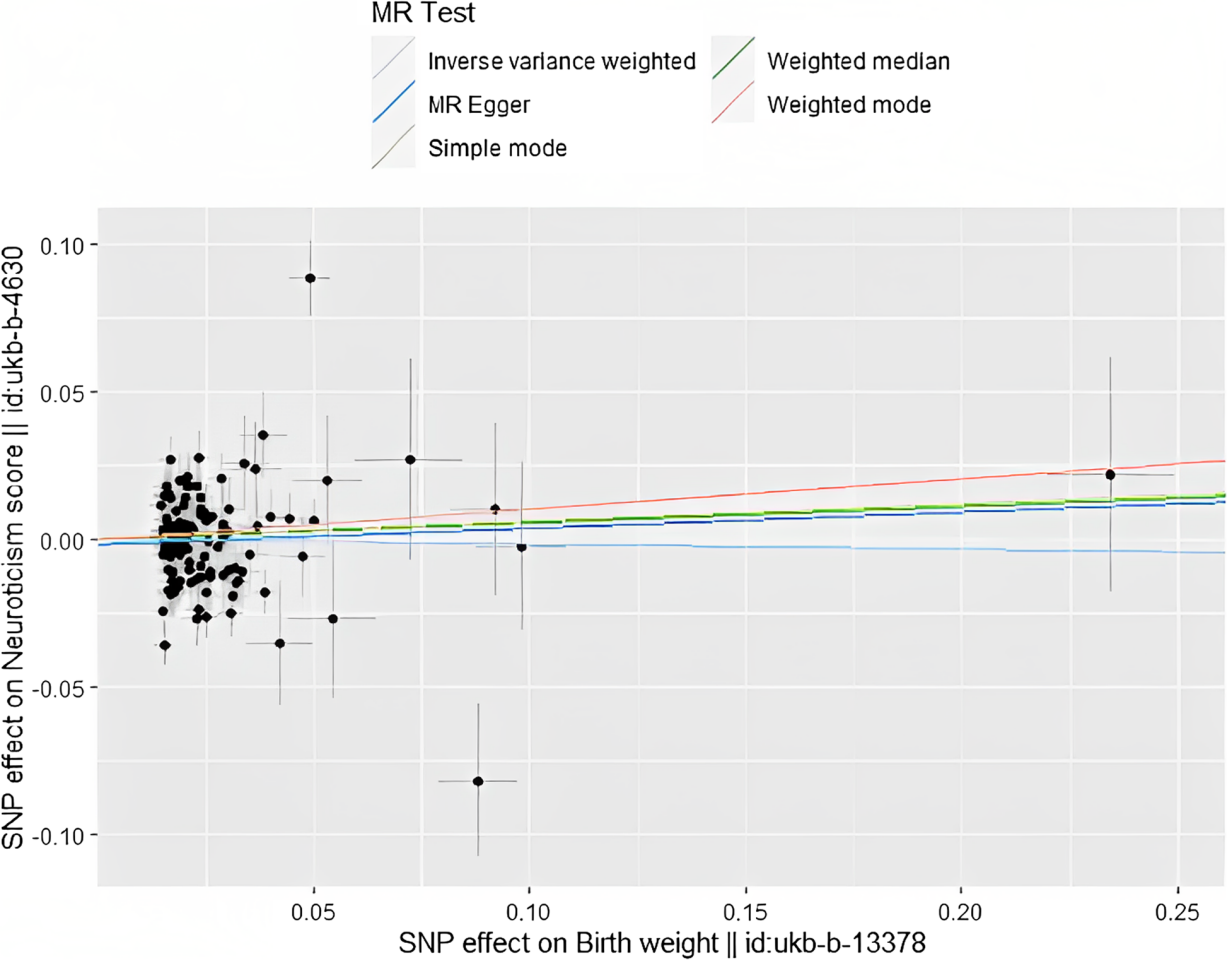
Scatter plot. SNP effect on BW

In the forest plot (Fig. 5), the red line in the bottom is the combined result, it reflects that the increase in BW does not increase the risk of neuroticism. Meanwhile, in the scatter plot (Fig. 6) it is not difficult to see from the plot that the risk of neuroticism has not obviously increased from the increase in BW. This result indicates that there is no obviously causal relationship.

## Discussion

This study analyzed the two independent datasets of neuroticism and BW based on GWAS by cFDR method to discover the potential SNPs related to neuroticism and BW. We identified 377 SNPs (cFDR < 0.01) significantly related to neuroticism, and 117 of them first discovered in our study. We also identified 337 SNPs (cFDR < 0.01) associated significantly with BW, which 52 of them are first discover in our study. Meanwhile, we have identified 62 SNPs with genetic pleiotropy common in neuroticism and BW. To eliminate the interference in confounding factors, we used data independent from the main study for validation analysis. The result of the validation analysis also shows the genetic pleiotropy, which further validated that there is a common genetic mechanism between neuroticism and BW.

Neuroticism is defined as a personality trait linked to emotional instability, which characterized by emotion dysregulation and negative affect. However, the pathogenesis of neuroticism is still unclear, in which genetic factors play an important role, and the current studies show that heritability of neuroticism ranged from 13 to 58% [13, 29]. Based on GWAS to search for susceptible genetic SNPs provides a new method for further revealing the genetic basis of the occurrence and development of neuroticism.

Notably, we identified 2 SNPs associated with neuroticism and BW (rs35755513 located on CSNK1G1/rs8039305 located on FURIN), which have both of eQTL and meQTL effect, and these SNPs might have significant effect on the pathogenesis of neuroticism and BW. CSNK1G1 located on chromosome 15, which is the member of the casein kinase I gene family. It encodes serine/ehreonine-protein kinase and mainly express in nervous system such as hippocampus, cerebellum and amygdala. Nina B. Gold's case report showed that CSNK1G1 associated syndromic developmental delay which all individuals have delayed growth, and possibly associated with autism spectrum disorder, facial features and seizure [30]. This case report enhanced the candidacy of CSNK1G1 as a cause of a neurodevelopmental disorder. Its related pathways are Wnt Signaling Pathway Netpath and Wnt Signaling Pathway, which Wnt signaling could glutamate-mediated rapid synaptic transmission [31–33]. Impairments of WNT signaling are known to underlie prenatal neuronal migration, thereby leading to developmental delay, abnormal behavioral and neurological symptoms [34].

FURIN also located on chromosome 15 and this gene encodes a member of the subtilisin-like proprotein convertases family. It encodes a type 1 membrane bound protease and mainly express in neuroendocrine. Among its related pathways are HIV Life Cycle and Lipoprotein metabolism, and the most associated diseases include Cerebral Amyloid Angiopathy, Itm2b-Related and Avian Influenza [35–37]. Furin is involved in the process of cleaving multiple protein precursors into mature proteins, such as brain-derived neurotrophic factor (BDNF). BDNF is a member of the neurotrophin family, and it plays an important role in neural differentiation, neural cell survival and synaptic plasticity [38]. A close relationship is found between neuroticism and increased BDNF gene methylation, which the higher the neuroticism score is related to higher level of BDNF gene methylation [39]. Therefore, abnormal process of protease FURIN cleaves proBDNF to generate mature BDNF (mbdnf) participate in neuropsychiatric disorders including neuroticism [40, 41].

In the functional exploration of pleiotropic gene, we also conducted GO enrichment analysis, finally we found “mesenchymal cell proliferation” and “DNA-binding transcription factor activity” are the most significant related pathways to neuroticism and BW. Mesenchymal stems cells may promote the proliferation and anti-apoptosis of human melanocytes through the PTEN pathway and DNA-binding transcription factor activity transcriptional regulatory activity that regulates genome transcription by selectively and non-covalently binding to specific double stranded genomic DNA sequences in cis regulatory regions, and it may relevant to chronic neurodegenerative diseases [42, 43]. DNA-binding transcription factor activity transcriptional regulatory activity that regulates genome transcription by selectively and non-covalently binding to specific double stranded genomic DNA sequences in cis regulatory regions. The SNPs related to neuroticism are mainly enrich in “glucose homeostasis pathway” and “type B pancreatic cell differentiation”, which has been researched in relation to insulin secretion, transport, gluconeogenesis, pathogenesis [44, 45], and several studies indicated that abnormal glucose homeostasis may contribute to serious mental illness [46]. Neural crests cell differentiation may relevant to mesenchymal structures of the brain, melanocytes and the peripheral nervous system [47]. As for the BW related SNPs, the GO result indicates that SNPs mainly enrich in “chromatin remodeling” and “transcription factor binding”.

MR analysis is more practical and effective than randomized controlled trials (RCT), which will not be easily affected by confounding factors and unclear causal timing [48, 49]. In these studies, we mainly used IVW methods to assess outcomes. It is not difficult to see that the effect size (b) of IVW is negative, indicating that an increase in BMI cannot lead to an increase in the risk of neuroticism. Combined with the results of the forest plot and scatter plot, we consider that they are less likely to be causal. It is not excluded that the result becomes negative due to the low statistical power caused by the removal of some SNPs in linkage disequilibrium or other residual confounders.

Our study has the following advantages. First, we used the cFDR and ccFDR method to analyze the functional SNPs associated with neuroticism and BW based on the latest large phenotype genetic dataset, and for the reason that cFDR has strong statistical power, we can realize more complex research design. Second, the common SNPs related to neuroticism and BW were discussed in our study, and the enrichment pathway and protein expression of related SNPs were also analyzed. In addition, we also performed eQTL and meQTL effect analysis on functional SNPs, which is conducive to identify candidate functional SNPs associated with neuroticism and BW and subsequent cellular and molecular biological functional studies. Third, we assessed the causal relationship between neuroticism and BW through MR analysis. Although the result suggests that the two traits have no causality, it does not mean that BW may not affect the occurrence of neuroticism through multiple intermediate variables. MR analysis was used to explore causal relationship. It refers to whether there is a causal relationship on the final outcome variable generated by the variant genetic locus. However, confounding factors such as the complex environment have no effect on genetic variation, which may lead to the difference between the results of RCT and MR.

Certainly, our study also has some limitation. First, due to the lack of individuals clinical data, we are unable to assess the impact of pleiotropic SNPs on traits, which limits the guidance on clinical practice. Second, in this study, our method of SNPs deletion is to delete the one with smaller MAF in a pair of SNPs with strong linkage, but such an approach may weaken the ability to identify rare key outliers. In addition, in the absence of further basic experimental verification, we intend to further verify the results of this study through molecular biological experiments.

In conclusion, we used the cFDR method to detect more potentially functional and significant pleiotropic SNPs of neuroticism and BW. We also estimated the causal relationship between neuroticism and BW through cFDR and MR analyses. Our finding provides a new idea for further understanding of potential shared genetic mechanism of neuroticism and BW and provides a reference basis for the diagnosis of neuroticism.

## Supplementary Information

**Additional file 1: Supplementary Table 1.** Conditional FDR value of 126 SNPs for neurotcism given the BW. **Supplementary Table 2.** Conditional FDR value of 148 SNPs for BW given the neuroticism. **Supplementary Table 3.** Results of the Validation Study: Conditional FDR value of 317 SNPs for neurotcism given the BW. **Supplementary Table 4.** Results of the Validation Study: Conditional FDR value of 70 SNPs for BW given the neuroticism. **Supplementary Table 5.** Results of the Validation Study: Conjunction cFDR value of 21 common SNPs in BW and neurotcism.

**Additional file 2.**

**Additional file 3.**

**Additional file 4.**

## Data Availability

The datasets analysed during the current study are available in the biobank repository.https://ctg.cncr.nl/documents/p1651/sumstats_neuroticism_ctg_format.txt.gz EGG (Early Growth Genetics) Consortium (egg-consortium.org). Trait: Neuroticism score—IEU OpenGWAS project (mrcieu.ac.uk). Trait: Birth weight—IEU OpenGWAS project (mrcieu.ac.uk).
